# *In vitro* testing of honey quality and biological functionality: underestimated elements in the clinical testing of honey

**DOI:** 10.3389/fnut.2024.1433786

**Published:** 2024-10-10

**Authors:** Juraj Majtan

**Affiliations:** ^1^Laboratory of Apidology and Apitherapy, Department of Microbial Genetics, Institute of Molecular Biology, Slovak Academy of Sciences, Bratislava, Slovakia; ^2^Department of Microbiology, Faculty of Medicine, Slovak Medical University, Bratislava, Slovakia

**Keywords:** quality standards, functional food, clinical trial, human health, natural product

## Abstract

Honey is an attractive functional food that often becomes a subject of clinical studies on the treatment of diverse diseases. However, the clinical efficacy of honey is rather controversial due, at least in part, to its variable composition and botanical origin as well as thermal processing or improper storage conditions. This review addresses the importance of honey quality standards and *in vitro* testing of the biological properties of honey prior to performing clinical studies, which can have a great impact on clinical outcomes. It focused on recently performed meta-analyses and systematic reviews where honey was used in the management of various disorders including respiratory tract infections, and metabolic and cardiometabolic diseases, with the goal of characterising the honeys used in clinical studies. In addition, it provides recommendations for the use and storage of honey for clinical testing. The vast majority of clinical studies included in meta-analyses do not provide any information about honey quality parameters. In fact, indicators of thermal damage or prolonged storage of honey were analysed only in one clinical study. This observation highlights on the alarming status of honey quality in clinical studies. Furthermore, *in vitro* biological properties of the analysed honeys were assessed in two clinical studies. Therefore, this review strongly advocates the clinical use of only fully characterised honey samples of known botanical origin with proven *in vitro* biological functionality and no or minimal thermal processing.

## 1 Introduction

Natural products from plants, microorganisms and insects are traditional sources of pharmacologically active compounds, especially in the area of novel antimicrobial drug discovery to address antimicrobial resistance (1). One of the major advantages of natural products is the synergic and poly-pharmacological effects their of active ingredients; however, these are difficult to prove experimentally (2). On the other hand, the low and highly variable content of biologically active ingredients in natural products represents the major limiting factor for their therapeutic assessment. The observed variability is due to the ambient environmental conditions, pre- and post-processing of natural products and their storage conditions (3). In addition, the absence of quality parameters that are correlated with the pharmacological activity of natural products often leads to a shortage of natural product-derived medicinal products.

Functional foods, defined as industrially processed or raw natural foods having potentially positive effects on health beyond basic nutrition, represent the largest group of natural products (4). However, to claim a certain food as a functional food, respectable experimental long-term safety evidence and proven clinical efficacy are essential. Clinical testing of functional foods is challenging and can be more complicated than conventional drug testing, especially in the case of double-blind, placebo-controlled, randomised trials (5). In addition, clinical trials provide information about functional efficacy but do not give insights into the mechanism(s) underlying it. Food functionality is based on the activity of ingredients possessing certain biological properties, including antioxidant, antibacterial and anti-inflammatory activities. These properties can be easily determined by *in vitro* tests but do not consider the complexity of physiological and biochemical processes in the human body (6). Therefore, preliminary *in vitro* screening of the biological activities of functional foods should be accompanied by *in vivo* testing in animal model and thus characterise the metabolic pathways driven by bioactive molecules in certain diseases.

The first essential step before any subsequent pre-clinical or clinical validation of functional foods is to evaluate their *in vitro* biological functionalities relevant to a specific disease. Some of the bioactive molecules in functional foods, such as polyphenols and proteins/enzymes, are characterised by poor stability and heat and light sensitivity, resulting in their degradation and loss of biological activity (7). Industrial processing and prolonged storage of functional foods may have a detrimental effect on the functionality of bioactive molecules.

Honeybee products have been used as remedies in traditional/folk medicine since ancient times. Honey, the best studied and most used honeybee product, has been considered not only as a sweetener but also as an attractive functional food (8). It has been the subject of numerous clinical studies to investigate its health benefits in the prevention and treatment of various diseases (9). In a substantial proportion of pre-clinical and clinical studies, honey was applied topically to treat various types of wounds. Positive clinical outcomes from topically applied honey enabled the creation of so-called ‘medical-grade honey’. Medical-grade honeys, used as medical devices, are prepared from certain types of honey which are subjected to sterilisation by gamma irradiation (10). In the case of medical-grade honey, rigorous standards must be met and comprehensive testing conducted to guarantee its safety and quality (10). However, most honey produced globally is classified as table honey for direct consumption, which does not need to fulfil strict criteria as medical-grade honey. Currently, honey quality criteria are based on the international honey standards, which are specified in the European Honey Directive (11) and in the Codex Alimentarius Standard for honey (12). Besides the fact that honey is a functional food, current legislative parameters do not consider the health-promoting biological properties of honey (9). A recent survey among honey consumers in the Balkans and Western European countries indicated that the main reason for honey consumption is its health benefits (13). Although honey is claimed as a functional food, results from clinical studies are rather inconsistent and conflicting, and it is difficult to draw comprehensive conclusions. There are several weaknesses associated with previously conducted clinical trials, and one of the systematically overlooked factors is the quality and functionality of the tested honey (14).

This study aims to provide perspectives and scientific opinions on honey as a therapeutic agent and to stimulate discussion about the quality issue with honey used in clinical studies. Furthermore, it proposes general recommendations for honey processing and storage conditions in which the biological functionality of honey is maintained over time.

## 2 International qualitative parameters of honey. Do they need an update?

Composition may differ from one honey to another and is affected by several factors, such as the botanical and geographical origin of the honey, environmental conditions and bee species. Apart from these factors, its composition can also be affected by beekeeper manipulation and processing (15). In order to guarantee honey safety and quality, a set of legislative qualitative standards has been adopted by Codex Alimentarius and the European Honey Directive (12) (Table 1). Due to natural differences in honey composition, the range of current honey standards is too wide, and numerous exceptions have been identified and codified in Codex Alimentarius for honey. Many differences exist and the majority of these differences are in moisture and sugar content and HMF content (15). Some countries adopted their own national provisions where more strict regulations are applied.

**Table 1:** General compositional criteria of honey with exceptions according to honey directive 2001/110 EU (11).

Criteria	Honey type		Exceptions (honey type)
	Blossom	Honeydew	
Moisture (%)	<20	<20	<25 (*Calluna* honey)
Sucrose (%)	<5	<5	<10 (*Robinia*, *Medicago*, *Banksia*, *Hedysarum*, *Eucalyptus*, *Eucryphia* spp., and *Citrus*) <15 *Lavandula* and *Borago*
Fructose and glucose content (sum of both %)	>60	>45	–
Electrical conductivity (mS/cm)	<0.8	>0.8	*Castanea*, *Arbutus*, *Erica*, *Eucalyptus*, *Tilia*, *Calluna*, *Leptosmermum* spp. and *Melaleuca*
DN	>8	>8	>3 (Honey with low natural enzyme content, e.g., *Citrus* honey: when HMF is less than 15 mg/kg)
HMF (mg/kg)	<40	<40	<80 (Honeys of tropical climate and their blends)

HMF, hydroxymethylfurfural; DN, diastase number.

Two of the legislative parameters, namely hydroxymethylfurfural (HMF) and enzymatic activity of α-amylase (diastase), which are related to honey freshness and its overheating during processing, are the most important parameters, especially for samples assigned for clinical testing. Numerous biologically active compounds are heat sensitive and tend to degrade over time. However, mounting evidence suggests that neither of these qualitative standards are always reliable parameters of marketed honey samples.

The legislated qualitative upper limit for HMF is 40 mg/kg, and its content in freshly collected honey is extremely low and highly unexpected. Although thermal treatment of honey increases the HMF content in treated honey, its concentration values are within the legislative limit in various types of honey, especially in stingless bee honey samples (16–20). Pasteurisation of pot honey at 65°C for 15 and 21 min as well as tyndallisation at 80°C for 15 and 21 min caused a significant increase in HMF formation, but the obtained values did not exceed the plausible limit for HMF (20). Similarly, the HMF content in stingless bee honeys exposed to high temperatures (between 75 and 95°C) for a short period of time (between 20 and 60 s) remained stable (16). In addition, extreme heating of stingless bee honeys at 90 and 95°C for 15 and 60 s did not change the initial HMF values in honeys before heating (17). These data indicate that thermal processing of stingless honey did not change the HMF level, most likely due to the presence of specific polyphenols acting as potential inhibitors towards the formation of harmful HMF (21).

Diastase and its enzymatic activity, expressed as diastase number (DN), are the sole qualitative parameters directly related to honeybee secretions from the hypopharyngeal glands. The enzymatic activity of diastase is more sensitive to thermal treatment in comparison with heat-induced formation of HMF. However, there is also evidence that the DN value does not change after thermal treatment (20, 22, 23) due to the ability of diastase to recover its enzymatic activity. The enzymatic activity of diastase can be naturally dramatically reduced in manuka honey type to DN values bellow permissible levels (24). Methylglyoxal, a major antibacterial compound in manuka honey, and 3-phenyllactic acid were found to accelerate the loss of diastase above that normally observed with time and elevated temperature. Likewise, citrus honey with a low natural enzyme content (DN value should be less than 3) and manuka honey should be treated as exceptions.

Another important weakness of DN as a qualitative parameter is based on the evaluation of enzymatic activity but not the origin of diastase. One of the current trends in honey adulteration is adding foreign diastase to disguise the enzymatic activity of bee-derived diastase (25–28). Almost 37% of honey samples from different regions in Türkiye (74 out of 202 samples) were diagnosed as having been adulterated by direct addition of foreign diastase or addition of syrup containing foreign diastase (27). This finding is alarming and represents the growing problem with honey authenticity worldwide.

Both DN and HMF content are important quality standards of honey; however, based on their above-mentioned weaknesses, it is desirable to assess more heat-sensitive parameters, such as enzymatic activity of bee-derived enzymes invertase (29) and glucose oxidase (30).

## 3 What is the status of clinical evidence for honey as a functional food?

Honey is a popular remedy worldwide and is still an attractive therapeutic product, especially in countries where healthcare systems might have low capacity, and patients mostly rely on traditional medicine. On the other hand, honey is also used in a modern medicine, especially as a medical-grade honey in wound care. Honey is a nutritional product where carbohydrates (95–97% of dry weight) represent the main composition. Fructose and glucose are the most important sugars of honey and contribute to most of the nutritional effects of honey (31, 32). Besides monosaccharides, honey contains small quantities of disaccharides (e.g., sucrose, galactose), trisaccharides (e.g., melezitose) and oligosaccharides which are mainly formed during honey ripening and maturation in bee colonies (31).

In the present work, searching for systematic reviews and meta-analyses was carried out, where honey was used as a functional food, and studies were published in scientific databases (PubMed, Scopus, and Web of Science) between 2019 and 2023. In the case of similar meta-analyses or systematic reviews describing identical health targets (e.g., cough, metabolic diseases), only the most recent one was included. Meta-analyses or systematic reviews using honey as a topical agent for the treatment of wounds, burns or oral mucositis were excluded. Furthermore, studies where honey was combined with other therapeutics or was part of natural product mixtures were not evaluated.

Four studies were identified where the effect of honey consumption on upper respiratory tract infections (URTIs) (33), acute cough in children (34), lipid profiles (35) and glycaemic control (36) was examined. Individual human clinical studies involved in selected meta-analyses/systematic reviews are listed in Table 2.

**Table 2:** Summary of data collection from human clinical studies using honey as a functional food.

Reference author country	Reference	Intervention	Comparator	Dose of honey	Honey qualitative parameters	Thermal processing of honey and storage conditions	*In vitro* testing of biological functionality of administrated honey
Respiratory tract infections and acute cough in children (33, 34)							
Ahmedi et al. (2013) Iran	(72)	Honey^*^	Diphenhydramine	N/A	N/A	N/A	No
Ayazi et al. (2017) Iran	(73)	1. Kimia honey^*^ 2. Shahde-Golha honey^*^	Diphenhydramine	2 × 2.5 mL (aged 1–6 yrs)/day 2 × 5 mL (aged 6–12 yrs)/day	N/A	N/A	No
Cohen et al. (2012) Israel	(74)	1. Eucalyptus honey (family Myrtaceae) 2. Labiatae honey (family Labiatae) 3. Citrus honey (family Rutaceae)	Silan date extract (placebo)	10 g/day	N/A	N/A	No
Paul et al. (2007) USA	(75)	Buckwheat honey^*^	1. Dextromethorphan 2. No treatment	1/2 teaspoon (aged 2–5 yrs) 1 teaspoon (aged 6–11 yrs) 2 teaspoons (aged 7–18 yrs)	N/A	N/A	No
Raeessi et al. (2011) Iran	(76)	Natural honey^*^	1. Natural honey with coffee 2. Coffee	3 × 25 g/day	N/A	Honey was applied in warm water (up to 60°C)	No
Shadkam et al. (2010) Iran	(77)	Natural honey^*^	1. Dextromethorphan 2. Diphenhydramine 3. No treatment	2.5 mL/day	N/A	Stored at RT	No
Waris et al. (2014) Kenya	(78)	Natural honey^*^	1. Salbutamol 2. Sugar syrup (placebo)	2.5 mL (aged 1–2 yrs) 5 mL (aged 2–6 yrs) 7.5 mL (aged 6–12 yrs)	N/A	N/A	No
Nishimura et al. (2022) Japan	(37)	Acacia honey	Artificial honey (placebo)	3 mL spoon/day	N/A	N/A	No
Glycemia (36)							
Abdulrahman et al. (2013) Egypt	(42)	Clover honey	No treatment	0.5 mL/kg body weight/day	N/A	Raw honey without heating and irradiation	No
Al-Walli (2004) UAE	(41)	Natural honey^*^	1. Dextrose 2. Artificial honey	75 g/250 mL water/day	N/A	Honey without any processing	No
Bahrami et al. (2009) Iran	(40)	Multifloral honey	No treatment	1 g/kg/day (first 2 weeks) 1.5 g/kg/day (second 2 weeks) 2 g/kg/day (third 2 weeks) 2.5 g/kg/day (last 2 weeks)	Moisture, F/G, sucrose content	Honey without any processing, stored at RT	No
Despland et al. (2017) Switzerland	(79)	Acacia honey	1. Control—low sugar 2. High fructose-glucose	25% of daily starch intake replaced by honey	Sugar analysis (fructose, glucose and sucrose content, F/G)	N/A	No
Enginyurt et al. (2017) Turkey	(71)	Honey^*^	No treatment	Three dosages of honey (5 g/day; 15 g/day and 25 g/day)	N/A	N/A	No
Husniati et al. (2013) Malaysia	(80)	Tualang honey	–	20 g/day	Honey was sterillised by gamma radiation (25 kGy)	N/A	No
Whitfield et al. (2016) New Zealand	(81)	Kanuka honey	Formulated kanuka honey (cinnamon, chromium and magnesium)	53.5 g/day	N/A	N/A	No
Yaghoobi et al. (2008) Iran	(82)	Natural honey^*^	Sucrose	70 g/250 mL water/day	N/A	N/A	No
Cardiometabolic diseases—lipide profiles (35)							
Al-Tamimi et al. (2019) USA	(44)	Clover honey (4 samples of different origin)	Sucrose	1.2 g/kg	Moisture, ash, protein, fat, total carbohydrates, sugar analysis (fructose, glucose, sucrose, maltose content)	One raw honey and three processed honey samples. All samples stored between 2–8°C.	No
Farakla et al. (2018) Greece	(46)	Thyme honey	Fruit marmalade	15 g/day	Sugar analysis (glucose and fructose)	N/A	TPC and antioxidative activity (ORAC assay)
Ghazali et al. (2017) Malaysia	(83)	Tualang honey	No treatment	20 g/day	N/A	N/A	No
Jayadi et al. (2019) Indonesia	(47)	Natural honey^*^	No treatment	70 g/day	N/A	N/A	Antioxidative activity (ORAC assay)
Majid et al. (2013) Pakistan	(43)	Acacia honey	No treatment	70 g/day	N/A	Honey without any processing	No
Münstedt et al. (2009) Germany	(45)	Polyfloral blended honey	Artificial honey	75 g/day	Moisture, sugar analysis (glucose and fructose), DN and HMF	N/A	No
Mushtaq et al. (2011) Pakistan	(84)	Natural honey^*^	No treatment	40 g/day	N/A	N/A	No
Raatz et al. (2015) USA	(85)	Blended honey	1. High-fructose corn syrup 2. Sucrose	50 g/day	Protein, fat, total carbohydrates, sugar analysis (fructose, glucose and sucrose content)	N/A	No
Rasad et al. (2018) Iran	(86)	Natural honey^*^	Sucrose	70 g/day	N/A	N/A	No
Rashid et al. (2019) Malaysia	(87)	Kelulut honey	No treatment	30 g/day	N/A	N/A	No
Tang et al. (2020) Malaysia	(69)	Tualang honey	No treatment	1. 20 g/day 2. 40 g/day 3. 60 g/day	N/A	N/A	No
Zakaria et al. (2018) Iran	(88)	Tualang honey	No treatment	20 g/day	N/A	N/A	No

* Botanical origin of honey is not available.

TPC, total polyphenol content; DN, diastase number; HMF, hydroxymethylfurfural; F/G, fructose/glucose; N/A, not available.

A recent meta-analysis of the clinical effectiveness of honey revealed that honey improves URTIs more effectively than the usual care in children, but only two of the included clinical studies were placebo controlled (33). In addition, honey was associated with a significantly greater reduction in cough frequency and severity. A very recent multicentre, placebo-controlled study conducted in paediatric patients with URTI showed a positive effect of acacia honey on nocturnal cough, but the observed effect did not differ from that of artificial honey, suggesting a role of sugar content in the antitussive effect of honey (37). The author of a recent systematic review on the efficacy of using honey to treat acute cough in children found a low quality of evidence that honey is effective in treating acute cough and improving sleep in children with acute cough (34). None of the analysed clinical studies involved in the meta-analysis (33) or systematic review (34) used honey that had been characterised in terms of qualitative legislative parameters (e.g., moisture, HMF, DN) nor *in vitro* biological functionality related to URTIs and cough (e.g., antimicrobial activity) (Table 1). Furthermore, in most clinical studies, no botanical and/or geographical origin was stated for the honey employed (Table 2).

Despite numerous pre-clinical studies reporting on the beneficial effects of honey on glycaemia, evidence regarding the effects of honey consumption on glycaemia in humans remains controversial, and no definite conclusion can currently be drawn (36). These controversies may be partly due to variations in study duration and in the type and dose of honey administered. The basic physicochemical characteristics (mainly the fructose and glucose contents) of administrated honey were determined in a few clinical studies but *in vitro* biological properties of honey related to glycemia were not analysed.

High intake of free or added sugars is one the major factors responsible for overweight, type 2 diabetes and cardiovascular disease development. In both adults and children, WHO recommends reducing the intake of free sugars, including honey, to less than 10% of total energy intake (38). However, honey has been considered as a natural remedy for diverse noncommunicable diseases and exerts numerous health benefits in pre-clinical and clinical studies. Two recently conducted systematic reviews and meta-analyses (35, 39) did not resolve the controversy surrounding the use of honey for improvement of the lipid profile, body weight and glycaemic control. Gholami et al. (39) demonstrated in the performed meta-analysis that honey consumption had no effect on serum lipids, including triglycerides, total cholesterol and low/high-density lipoprotein cholesterol. On the other hand, Ahmed et al. (35) showed in conducted meta-analysis that oral honey intake reduced fasting glucose and triglycerides, total cholesterol and low-density lipoprotein cholesterol. Interestingly, the authors demonstrated that some types of honey, such as clover and acacia honey, exhibited the beneficial effect of lowering glycaemic and cholesterols values. Likewise, raw and/or unprocessed honey showed higher efficacy than the processed variety. This observation highlights the importance of botanical origin and processing when selecting a honey type for clinical use. Raw, unprocessed honey of known botanical origin with certain proven biological properties should be used in clinical studies.

In total, 5 out of the 28 clinical studies (40–44) listed in Table 2 used raw, unprocessed honey for clinical testing. Furthermore, DN and HMF values, as indicators of honey overheating or prolonged storage, were determined only in one study (45). The biological activity, namely antioxidant activity, of honey was evaluated in two studies (46, 47) (Table 2). These findings indicate that the vast majority of clinical studies conducted ignored qualitative parameters of honey. In addition, the authors were not aware of the importance of the biological functionality of honey and the necessity of *in vitro* testing prior to clinical testing.

## 4 Qualitative and biological parameters of tailored honey for initial screening of its functionality

Although the qualitative parameters of honey are widely established worldwide, none of them consider the health-promoting effects of honey or its biological properties. Moreover, as mentioned above, cumulative scientific evidence indicates certain weaknesses of qualitative parameters, particularly in honey processing and handling.

Raw or unprocessed honey seems to have some advantages compared to thermally processed honey; however, it is necessary to minimise the risk of fermentation during storage and avoid spoilage. Honey is a natural product which may contain a great variety of bacteria, yeasts and fungi. Some bacteria and fungi encountered in honey have been associated with human diseases and although the health risk to humans is low in adults (48), it is essential to assess the microbiological quality of honey.

This review strongly advocates the adoption of basic honey qualitative parameters according to Codex Alimentarius (12) by researchers/clinicians who are planning to conduct human clinical studies where honey will be used as a potential therapeutic product. Researchers should pay particular attention to the HMF and DN values of tested honey samples. Additionally, it is essential to evaluate the biological functionality of tested honey samples before their use in a clinical study. There are several well-described biological properties of honey, such as antimicrobial/antibiofilm, antioxidant and anti-inflammatory activity, which might be suitable for *in vitro* honey testing. The selection of an *in vitro* assay depends on the subject of the clinical study to be investigated.

### 4.1 Antimicrobial activity of honey

Antimicrobial activity is the most studied biological property of honey, and recently it has been proposed as a suitable qualitative parameter of honey (9). The antibacterial activity of honey is determined by several different factors and molecules, such as low pH, high sugar content (osmolarity), hydrogen peroxide (H_2_O_2_), methylglyoxal and the bee antibacterial peptide defensin-1. Furthermore, certain types of phytochemicals, especially polyphenolic compounds, may elevate the antibacterial effect of honey (49–51). A detailed characterisation of the antibacterial effect of honey allows effective use of topical honey-based formulations and medical-grade honeys in wound care.

A range of testing methods are used to determine the antimicrobial activity of honey against diverse bacteria and fungi. Nowadays, it is very demanding to compare the overall antibacterial efficacy of various types of honey due to differences in the assays employed. Among testing methods, the agar diffusion and broth dilution method are the most often used worldwide (52). Since honey is a complex of diverse compounds with different molecular weights, the agar diffusion method has some limitations and can generate inaccurate outcomes. Therefore, the broth dilution method seems to be the most suitable method of determining the antibacterial potential of honey regardless of the botanical and geographical origin of analysed honey. The antibacterial activity of honey is typically expressed as the minimum inhibitory concentration (MIC) or minimum bactericidal concentration (MBC). However, serious discrepancies were found in the broth dilution method where different experimental conditions (e.g., static vs. shaking, doubling dilution vs. small increments, visual vs. optical inspection) were used. This review advocates following the methods published by the Clinical and Laboratory Standards Institute (CLSI) (53) or European Committee for Antimicrobial Susceptibility Testing (EUCAST) (54), with some modifications for honey testing. A robust, standardised, cost-effective method of determining the antibacterial potential of honey is urgently needed. The antibacterial activity of honey can be significantly reduced after thermal processing (55, 56) or improper storage (57, 58), whereas values of the traditional quality parameters HMF and DN can be within the range of permissible levels.

### 4.2 Antioxidant activity of honey

Investigation and characterisation of antioxidant activity in honey is an attractive research area. Honey is deemed a potential source of natural antioxidants that can counteract the effects of oxidative stress underlying the pathogenesis of many diseases. Several components of honey are responsible for antioxidant activity, including polyphenolic compounds (phenolic acids and flavonoids), vitamin C and E, enzymes (catalase and peroxidase), Maillard reaction products and trace elements (59, 60). The concentration of these bioactive compounds is highly variable and depends on the type of flora, geographical location of production, climatic conditions, seasonal factors and soil composition, as well as the production process (61).

Measurement of the antioxidative potential of natural products is not based solely on one method; rather, several different types of methods (e.g., spectrometric, electrochemical and chromatographical assays) should be employed (62). There is no official method for determination of the antioxidant activity of honey, and none of the methods used are ideal, each being designed to measure a different group of antioxidants (61). The antioxidant activity of honey is generally measured in the form of antiradical activity using the 1,1-diphenyl-2-picrylhydrazyl (DPPH) scavenging assay, 2,2′-azino-bis (3-ethylbenzothiazoline-6-sulphonic acid) (ABTS) assay, oxygen radical absorbance capacity (ORAC) assay and ferric reducing antioxidant power (FRAP) assay (63).

The total phenolic content and antioxidative activity of honey can be greatly affected by its processing and storage. Uneven changes in the antioxidant activity of thermally processed honey have been observed. Thermal processing is able to either decrease or increase the antioxidative potential of a particular honey sample [reviewed in Faraz et al. (64)]. The changes in the antioxidant activity of honey depend on two major factors: honey composition and the temperature and duration of thermal treatment (65). On the other hand, the antioxidant capacity of honey after thermal processing also depends on the method(s) used to evaluate this activity. Considering the fact that honeys of different botanical origin are clinically tested, this review proposes that thermal processing or prolonged storage of honey be avoided, particularly in cases where oxidative stress plays a role.

### 4.3 Anti-inflammatory activity of honey

Inflammation is a key factor in the development of chronic diseases, including cardiovascular diseases and diabetes. Honey is considered as an anti-inflammatory agent which may suppress the production of mediators of inflammation (e.g., pro-inflammatory cytokines) and support haemostasis (32). Various *in vitro* models of inflammation have been used to characterise the anti-inflammatory effect of honey.

The composition of honey precludes the testing of honey itself due to its effect on the cultivation medium. Therefore, the individual components of extracted or purified honey, particularly polyphenolic compounds, are tested for anti-inflammatory activities. The cell types most often used for *in vitro* screening are primary cultures of human cells and human cell lines such as peripheral blood mononuclear cells, RAW 264.7 macrophages, HaCaT cells and human THP-1 monocytes (66).

Honey components act as (i) modulators of pro-oxidant enzymes (cyclooxygenase-1 and -2 inhibition, nitric oxide synthase and lipoxygenase inhibition), (ii) modulators of pro-inflammatory mediators (TNF-α, IL-1 and Il-6 production and nitrate/nitrite measurement) and (iii) modulators of nuclear factor expression (*NF-kB*) (*p65*NF-kB levels) (66).

Literature is scarce regarding characterisation of the effect of industrial processing including thermal treatment or prolonged storage on the anti-inflammatory activity of honey. Similar to the antioxidative effect, it is assumed that thermal treatment affects anti-inflammatory activity in the same way, since polyphenols are responsible for the observed *in vitro* anti-inflammatory effect.

## 5 Recommendations for honey use in clinical medicine

As stated above, honey is an attractive therapeutic agent for both topical and oral use. According to the web resource www.clinicaltrails.gov (date: January 26, 2024; terms: honey), seven human clinical trials using honey as a dietary supplement are currently active or in recruiting mode focusing on periodontal disease, postprandial glycemia, poor quality sleep, oxidative stress, xerostomia, overweight and insulin resistance.

**Figure 1:**
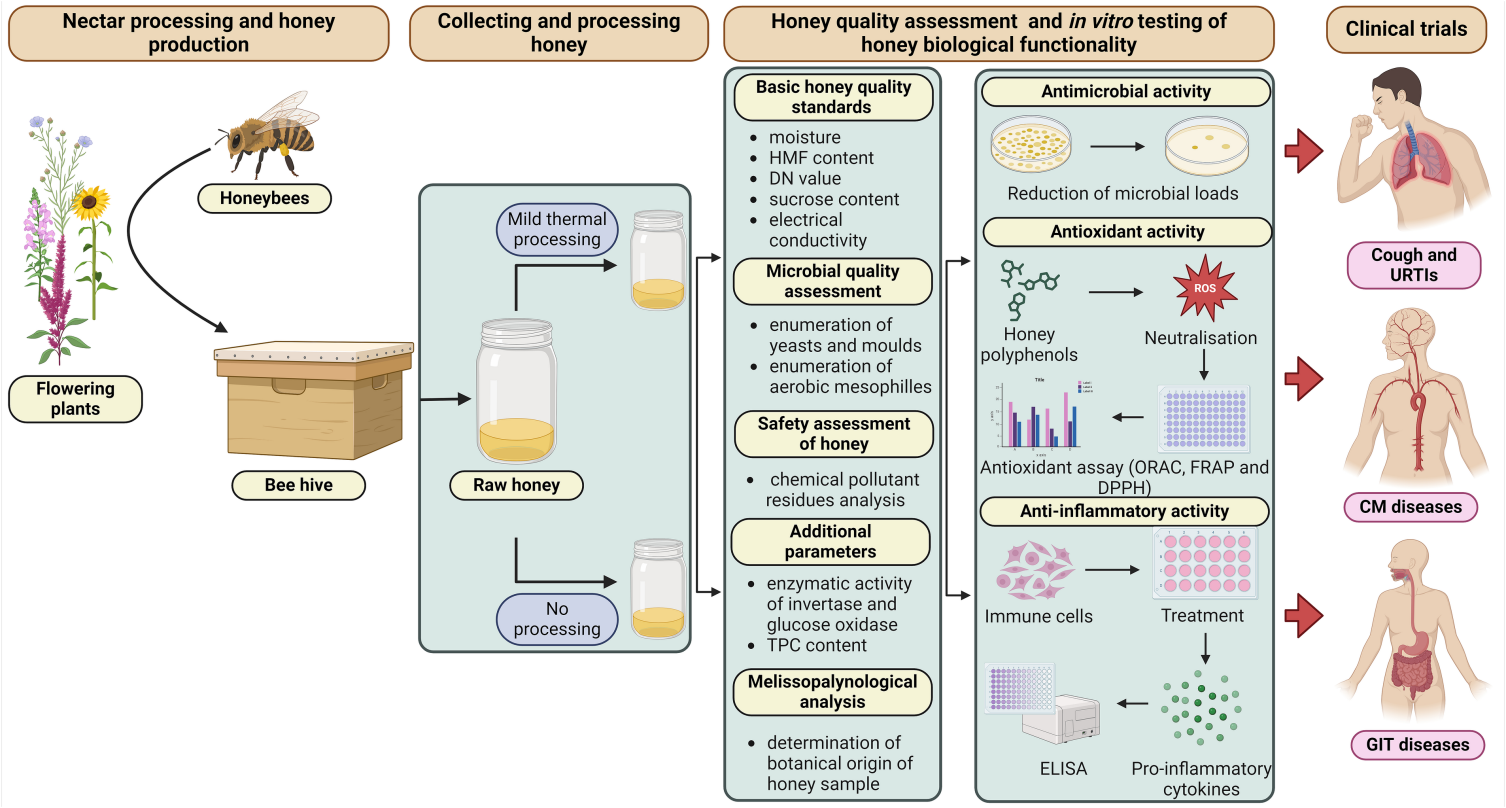
Ideal and recommend practises for honey processing and *in vitro* testing of its qualitative parameters and biological activities. Created with BioRender.com.

This review recommends that only honey of known botanical/geographical origin, with fully characterised qualitative parameters and, if possible, without thermal processing should be used for clinical testing (Figure 1). In addition, it is also strongly recommended to verify its biological functionality using an *in vitro* assay, depending on the disease/condition to be treated.

**Figure 2:**
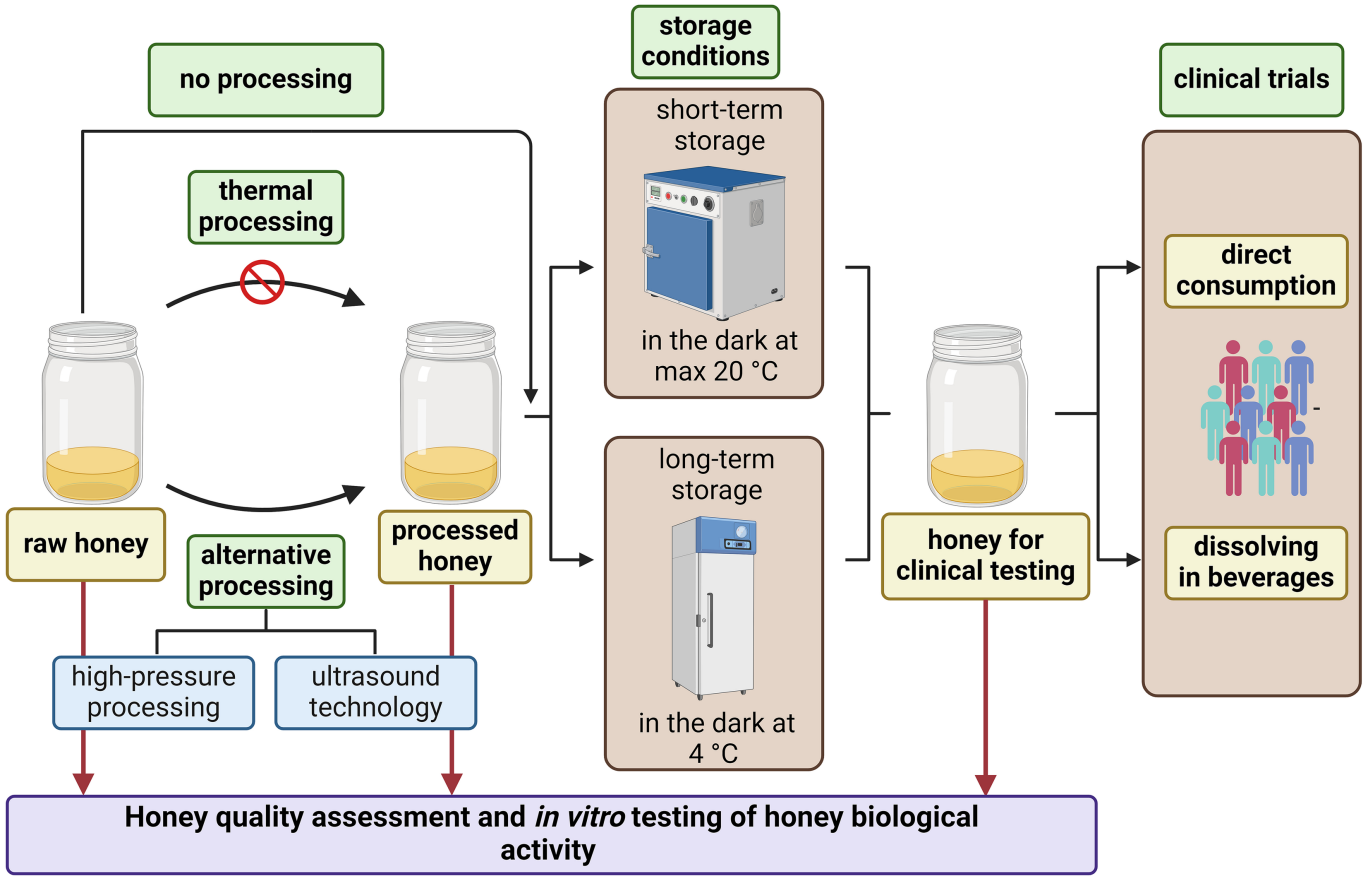
Recommendations for honey processing and its use as oral therapeutic agent in clinical medicine. Created with BioRender.com.

In order to test for a safe honey without harmful microorganisms or if reduction of the honey microbial load is needed, alternative methods of honey preservation, other than thermal treatment, is recommended (Figure 2). Nowadays, due to unfavourable side-effects of thermal pasteurisation, other approaches such as ultrasound and high-pressure processing are being tested to avoid honey spoilage and fermentation whilst maintaining health-promoting properties (67, 68).

Regarding the storage of tested honey samples, it is recommended that honey samples be kept at temperatures up to 20°C (preferably at 4°C) in a dark place (Figure 2). If the study design requires dissolution of honey in water or other beverages, it is recommended that they be used at room temperature and, if possible, that warm/hot beverages be avoided. Honey or honey in beverages should be consumed on an empty stomach, thus avoiding potential unwanted interactions with other food/food supplements.

One of the most debatable issues is the optimal consumed dose of honey to use in clinical studies. There have been several studies that tested different doses of honey in the clinical setting (69–71). Although different doses of honey were tested, no significant changes in honey efficacy were found. However, the optimal dose of consumed honey for clinical studies together with the duration of administrated honey must be established. On the other hand, precautions are needed for high-dose honey supplementation, as it may cause increased blood glucose levels.

## 6 Conclusion

Controversial outcomes of clinical trials that used honey as a functional therapeutic agent in a broad spectrum of diseases are becoming the object of debate. Differences in honey composition have been suggested as major explanations for opposite clinical outcomes. However, it is showed here that honey quality and biological functionality are the key parameters which may significantly determine the clinical efficacy of honey in clinical trials. According to the evaluated meta-analyses and systematic reviews, both parameters are deeply underestimated by clinicians. Furthermore, storage conditions and/or thermal processing may negatively affected honey quality and biological functionality and these facts need to be taken into account before conducting clinical studies. Therefore, the review strongly advocates the clinical use of only fully characterised honey samples of known botanical origin with proven *in vitro* biological functionality and no or minimal thermal processing.
